# Occupational class differences in daily sitting time among young and early midlife public sector employees—a follow-up study

**DOI:** 10.1093/eurpub/ckag110

**Published:** 2026-06-24

**Authors:** Ville Päivärinne, Eero Kekäläinen, Olli Pietiläinen, Jatta Valkonen, Jouni Lahti, Eero Lahelma, Anne Kouvonen, Ossi Rahkonen, Tea Lallukka

**Affiliations:** Department of Public Health, University of Helsinki, Helsinki, Finland; Finnish Institute for Health and Welfare, Helsinki, Finland; Department of Public Health, University of Helsinki, Helsinki, Finland; Department of Public Health, University of Helsinki, Helsinki, Finland; Department of Public Health, University of Helsinki, Helsinki, Finland; Department of Public Health, University of Helsinki, Helsinki, Finland; Finnish Institute for Health and Welfare, Helsinki, Finland; Department of Public Health, University of Helsinki, Helsinki, Finland; Faculty of Social Sciences, University of Helsinki, Helsinki, Finland; Centre for Public Health, Queen’s University, Belfast, Northern Ireland; Department of Public Health, University of Helsinki, Helsinki, Finland; Department of Public Health, University of Helsinki, Helsinki, Finland

## Abstract

Longitudinal and domain-specific changes in sitting time (ST) across occupational classes are poorly understood. We examined changes in occupational class differences in total and domain-specific ST over a 5-year follow-up among young and early midlife employees. We used Helsinki Health Study survey data from 2017 to 2022 (*n = *2762; 81% women; aged 19–39 years). Self-reported ST (min/day) was assessed across total ST and its five subdomains (work, leisure screen time, reading, transport, and other). Occupational class was categorized as manual and routine non-manual, semi-professional, and professional. Linear mixed models were used to estimate changes, with ST as the dependent variable and time, occupational class, and their interaction as the main independent variables. Analyses were stratified by gender and adjusted for sociodemographic and health-related factors. Total ST increased across occupational classes (+46 min/day, 95% CI: 37.6–54.4), driven by work-related ST (+24 min), and leisure screen ST (+21 min). Among women, manual and routine non-manual employees showed larger increases in total ST (+25 min vs. professionals) and leisure screen ST (+14 min), narrowing the total ST but widening screen time occupational class differences. Semi-professional women increased work ST (+21 min vs. professionals), narrowing occupational class differences in work ST. Among men, statistical significance of the associations could not be confirmed. ST increased across occupational classes, with domain-specific changes suggesting behavioural patterns. Increase in leisure screen time among manual and routine non-manual women employees may contribute to socioeconomic inequalities in health, highlighting the need for targeted, domain-specific interventions.

## Introduction

Sedentary behaviours refer to any waking behaviour characterized by an expenditure of ≤1.5 metabolic equivalents while in a sitting, reclining, or lying posture [1]. It is typically measured through self-reported sitting time (ST), accelerometer data, or commonly used markers, such as television viewing. Prolonged sedentary time has been associated with various adverse physical and mental health outcomes, including cardiovascular disease, type 2 diabetes, certain cancers, and all-cause mortality [1–4]. Beyond total ST, the domains in which sedentary behaviour occurs—such as during work, leisure screen time, transportation, and other contexts—may also have distinct health implications [5].

Previous research has consistently shown that socioeconomic position—particularly occupational class—is associated with patterns of sedentary behaviour [6]. Individuals in higher occupational classes often report more total ST, largely driven by work-related sitting, while those in lower occupational classes tend to accumulate more ST during leisure time [7]. However, while cross-sectional associations between occupational class and sedentary behaviours are relatively well-documented, there is a lack of longitudinal research examining how changes in ST over time vary across occupational classes [8]. Previous longitudinal population-based studies have shown mixed trends in sedentary time, with both increases and decreases across populations, often associated with occupational class, individual variability, and more recently, the COVID-19 pandemic [9–13]. Moreover, higher body mass index (BMI) [8], low physical activity [14], and insufficient sleep [15] have been associated with increased ST. These variables are therefore important to consider, when examining changes in ST by occupational class.

Understanding how ST, both total and domain-specific, changes over time among different occupational classes is important, as increasing ST may further widen socioeconomic disparities in health. Moreover, most previous studies have primarily focused on total ST, with less attention to simultaneous domain-specific changes, such as sitting during work, leisure screen time, reading and transportation [11]. Considering domain-specific ST provides a more nuanced understanding of how different socioeconomic groups accumulate ST and how these patterns evolve over time. These patterns can be understood within the broader framework of persistent health disparities associated with socioeconomic class structures [16].

This study aimed to examine how occupational class is associated with total and domain-specific ST, including work, leisure screen time, leisure reading, transportation, and other domains, and their changes among young and early midlife employees over a 5-year follow-up.

## Design and participants

The survey was conducted in autumn 2017 among employees of the City of Helsinki, Finland, aged 19–39, years who were eligible at the time of sampling and had an employment contract of at least 50% for a minimum of four months prior to sampling (*n = *11 459). Data were gathered using online questionnaires (58%), postal surveys (29%), and telephone interviews (13%) [17]. Participants with a registered email address were invited to complete the survey online, while paper questionnaires were mailed reflecting the gender distribution of the target population [17]. Consent to register linkage (81%, *n = *4864) was required for inclusion in the study population. Participants who were interviewed by telephone (*n = *651) were excluded, because the interview did not cover the variables of interest to this study. The Phase 2 survey, collected in 2022 via online, mail, and phone, was sent to all Phase 1 participants (*n = *3520, response rate 60%). Participants who completed the survey via telephone interviews (*n = *285) were excluded, as ST and other variables of interest were not collected through this method. To ensure data completeness, we included only participants with no missing values in ST in Phase 1 and Phase 2 and occupational class in Phase 1. This resulted in a final analytical sample of 2762 participants, of whom 81% were women (Supplementary File 1). Survey questions used in this study are provided in Supplementary File 2. The study protocol has been approved by the ethics committees of the Faculty of Medicine, University of Helsinki, and the authorities of the City of Helsinki.

More detailed description of the data and the participants can be found elsewhere [17, 18].

### Measures of sedentary time

Sedentary behaviour was assessed based on participants’ self-reported average ST on a typical weekday across five domains: watching TV or using a computer at home, reading at home, time spent in a vehicle, sitting during work hours, and time spent sitting in other locations. These categories correspond to domains commonly used in previous research [9, 19]. The distribution of ST data was slightly skewed to the right, leading to non-normally distributed residuals in the models. To mitigate this, extreme values were identified and excluded. Outliers were defined as individuals reporting more than 18 hours of total sitting per day, based on a z-score threshold of 3 [20]. Following this procedure, 97 participants were excluded from the analysis.

### Occupational class

Occupational class was derived from the job title sourced from the employer’s personnel register for those who gave their written informed consent for the linkage, and for other participants, from the Phase 1 survey. Occupational class was classified into three categories: ‘manual or routine non-manual workers’ (e.g. cooks, firefighters, childcare workers, and practical nurses), ‘semi-professionals’ (e.g. registered nurses, and early years teachers) and ‘professionals’ (e.g. primary or secondary school teachers, social workers and counsellors, general practitioners, and project managers). A more detailed description of the most common occupations in each occupational class by gender can be found in the Table S1.

### Covariates

As covariates, we included key factors that have been associated with ST and occupational class in previous studies [8]; that is age, marital status, work status, body mass index (BMI), leisure-time physical activity (LTPA), sleep sufficiency, self-rated health, all derived from the Phase 1 survey.

Age was first derived from each participant’s birth year and then centred by subtracting the sample mean age (32.4 years). The resulting variable reflects each individual’s deviation from the mean age in the study population. Marital status was divided into married/cohabiting vs. other (i.e. single, divorced, or widowed). Work status was categorized to differentiate participants who were temporarily outside the labour market (e.g. due to studying, parental leave, or long-term sickness absence) from those actively working. LTPA was categorized into three groups according to metabolic equivalent (MET) hours: low or moderate activity (<20 MET-hours per week), vigorous activity (20–60 MET-hours per week, including vigorous activity), and high vigorous activity (>60 MET-hours per week, including vigorous activity). Sleep sufficiency was categorized based on the respondent’s answer as sufficient (‘almost always’ or ‘often’) or insufficient (‘rarely’ or ‘hardly ever’). Participants rated their overall health on a 5-point scale ranging from ‘poor’ to ‘excellent.’ We combined ‘excellent,’ ‘very good,’ and ‘good’ into a good health category, and ‘fair’ and ‘poor’ into a poor health category.

### Statistical analyses

Background characteristics of the participants are presented as means (continuous variables) or percentages (categorial variables). Group differences in categorial variables were assessed with the Chi-squared test and analysis of variance (ANOVA) was conducted for continuous variables.

We used linear mixed models (LMM) to analyse changes in ST. Changes were reported in minutes per day, along with their 95% confidence intervals (CIs). Based on prior evidence that men and women differ in domain-specific sedentary patterns [21], we stratified all analyses by gender. Stratification therefore allowed us to fully capture and report these established gender-specific patterns. All ST domains used these full samples, except the work domain, which included only those who were at work at both phases (women, *n = *1662; men, *n = *479). If a participant had not provided ST in one domain but had provided it in other domains, the ST for the missing domain was assigned a zero value. In the unadjusted model, minutes across both phases were used as a dependent variable, separately for different sedentary domains, and occupational class was used as an independent variable as a fixed effect. A subject-specific intercept was included as a random effect. This model gives estimates for the baseline ST and the change in it. In the base model, age was added to the unadjusted model as a fixed effect along with occupational class and its interaction with phase, thereby adjusting for the effects of the independent variable on the baseline ST and the change in it separately.

In the LMM model, parameter estimates were presented as β with their 95% CIs to provide estimates of changes in ST, expressed in minutes. Furthermore, marginal effects with their 95% CIs were estimated, with average marginal effects (AMEs) representing absolute changes in sedentary minutes. Because multiple domain-specific sedentary behaviour outcomes were analyzed in parallel, Šidák correction [22] was applied to account for multiple comparisons and to reduce inflation of type I error. The Šidák method was selected as a less conservative alternative to Bonferroni correction while maintaining control over the family-wise error rate. Stata 19.5 (StataCorp LP; College Station, TX, USA) statistical package was used for all analyses.

## Results

In the study population at Phase 1 (Table 1), women who were not married or cohabiting had ∼33 min/day more ST than those who were married or cohabiting (367 ± 191 min/day vs. 400 ± 192 min/day, *P* = .001). Being at work in Phase 1 was associated with higher ST in both genders (*P* < .001). In women, those with obesity spent ∼38 min/day more ST than those with normal weight. In addition, insufficient sleep (∼21 min/day, *P* = .017) and poor SRH (∼29 min/day, *P = .*028) were associated with more ST among women. In women, higher occupational class was associated with substantially more ST; professionals showed ∼62 min per day more ST than manual or routine non-manual workers. For men, a statistically significant difference in ST was found only for work status. Women and men differed most in work and screen time domains, with men having more ST in those two domains.

**Table 1.** Descriptive characteristics and mean daily sitting time (ST) minutes in 2017 (Phase 1) by gender (women *n = *2233; men *n = *529) among the Helsinki Health Study participants.^a^

	Women (*n*, %)	ST (mean, SD)	*P*	Men (*n*, %)	ST (mean, SD)	*P*
	2233 (100)			529 (100)		
Age			.87			.51
19–29	642 (29)	376 (194)		126 (24)	467 (203)	
30–34	774 (35)	377 (192)		164 (31)	460 (202)	
35–39	817 (36)	381 (191)		239 (45)	443 (204)	
Marital status			.001			.27
Married or cohabiting	1444 (65)	367 (191)		147 (28)	469 (216)	
Other	789 (35)	400 (192)		382 (72)	448 (192)	
Work status			<.001			<.001
Working	1960 (88)	396 (192)		510 (96)	461 (202)	
Not working	273 (12)	247 (136)		19 (4)	255 (110)	
Body mass index			.002			.49
<25.0 kg/m^2^	1389 (62)	368 (188)		243 (46)	453 (200)	
25.0–29.9 kg/m^2^	512 (23)	388 (193)		201 (38)	445 (208)	
≥30.0 kg/m^2^	332 (15)	406 (204)		85 (16)	476 (198)	
Sufficient sleep			.017			.15
Sufficient	1482 (66)	371 (191)		372 (70)	446 (194)	
Insufficient	751 (34)	392 (194)		157 (30)	473 (222)	
Leisure-time physical activity			.34			.10
High vigorous	681 (31)	372 (184)		219 (42)	433 (192)	
Vigorous	879 (39)	385 (196)		198 (37)	476 (200)	
Low/moderate	673 (30)	375 (195)		112 (21)	456 (226)	
Self-rated health			.028			.28
Good	1989 (89)	375 (191)		459 (87)	450 (199)	
Suboptimal	244 (11)	404 (201)		70 (13)	478 (224)	
Occupational class			<.001			.06
Professional	625 (28)	407 (183)		174 (33)	461 (194)	
Semi-professional	992 (44)	381 (197)		154 (29)	479 (198)	
Manual/routine non-manual	616 (28)	345 (189)		201 (38)	428 (212)	
Sedentary time						
Total	2233 (100)	378 (192)		529 (100)	454 (203)	
During work	1662 (74)	200 (141)		479 (91)	214 (142)	
Leisure screen time	2233 (100)	115 (80)		529 (100)	145 (85)	
Leisure reading	2233 (100)	37 (43)		529 (100)	35 (41)	
Transportation	2233 (100)	57 (40)		529 (100)	56 (62)	
Other	2233 (100)	21 (46)		529 (100)	24 (44)	

a *P* value denote within-gender differences in ST across each covariate, based on independent *t*-tests for dichotomous variables and one-way ANOVA for variables with more than two categories.

**Figure 1.**
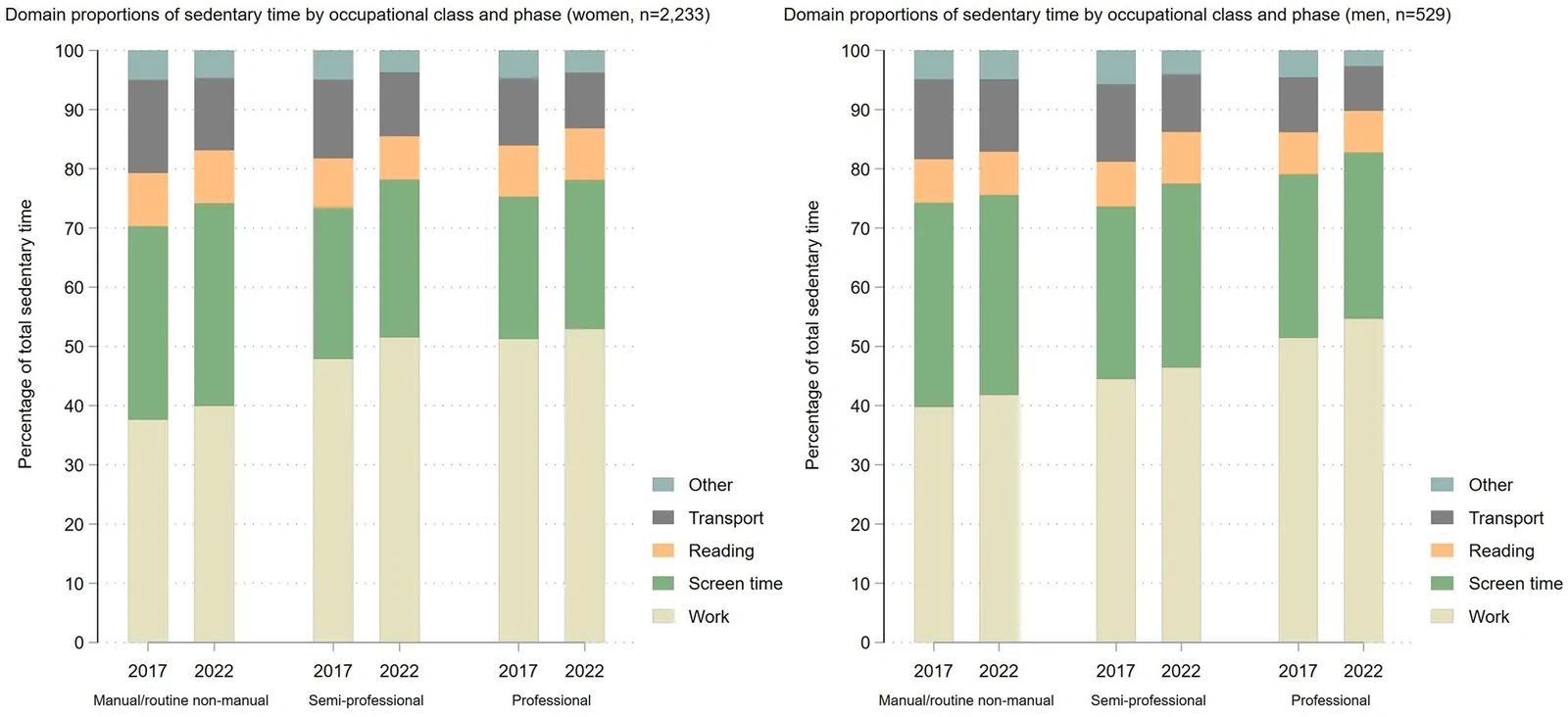
Relative daily sedentary time (%) across five domains by occupational class among the Helsinki Health Study participants (Phase 1: 2017; Phase 2: 2022). Women (*n = *2233) and men (*n = *529) are shown separately.

Figure 1 shows how total ST was distributed across the ST domains in 2017 compared to 2022 for each occupational class. In both women and men, work-related ST made up the largest share, rising steadily from manual or routine non-manual to professional groups, and increasing slightly over the 5-year period. By contrast, in the lowest occupational class leisure screen ST comprised the largest share and also increased slightly over the same period. There was a slight decrease in transportation-related ST in each occupational class over time.

**Figure 2.**
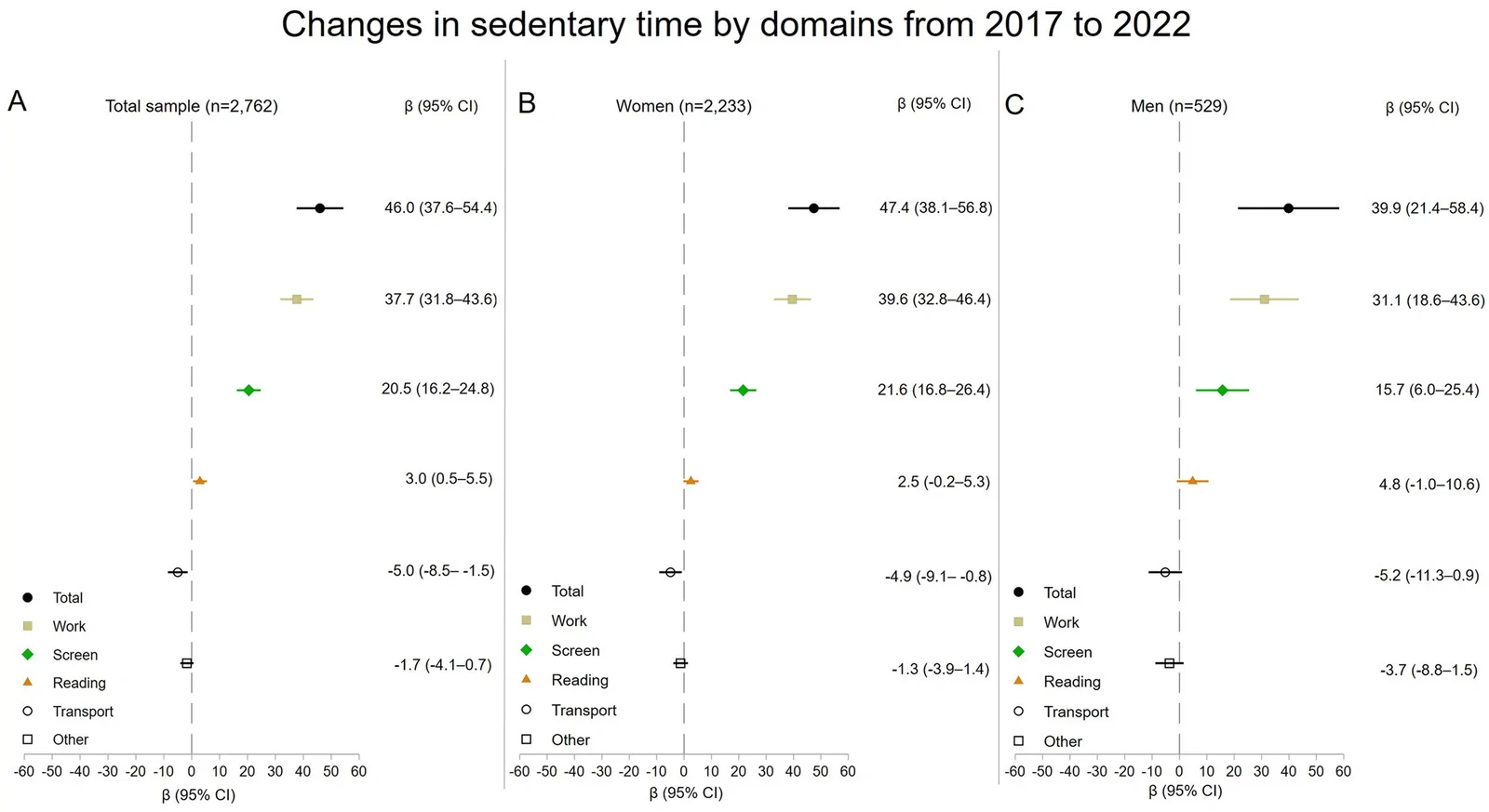
Changes in total and domain‐specific sedentary time (minutes/day) from 2017 to 2022 by domains and gender among the Helsinki Health Study participants; ‘A’ = total sample (*n = *2762), ‘B’ = women (*n = *2233), ‘C’ = men (*n = *529). Linear mixed‐effects models (random intercept for participant) estimated β‐coefficients (95% CIs). All models are adjusted for age.

During the 5-year follow-up (Fig. 2), daily total ST increased across the study population, accompanied by increases in work-related and leisure screen ST, while transportation-related ST decreased. Daily total ST increased (β = 46 min, 95% CI: 37.6–54.4) in overall sample (*n = *2762), as did ST during work (β = 24 min, 95% CI: 18.1–29.7), leisure screen ST(β = 21 min, 95% CI: 16.2–24.8), and leisure reading ST (β = 3 min, 95% CI: 0.5–5.5), whereas ST in transportation decreased (β = −5 min, 95% CI: −8.5 to −1.5). Total ST increased for both genders (women: β = 47 min, 95% CI: 38.1–56.8; men: β = 40 min, 95% CI: 21.4–58.4), as well as for work-related ST (women: β = 40 min, 95% CI: 32.8–46.4; men: β = 31 min, 95% CI: 18.6–43.6), screen time ST (women: β = 22 min, 95% CI: 16.8–26.4; men: β = 16 min, 95% CI: 6.0–25.4). In addition, women decreased their transport-related ST (β = −4.9 min, 95% CI: −9.1 to −0.8).

**Figure 3.**
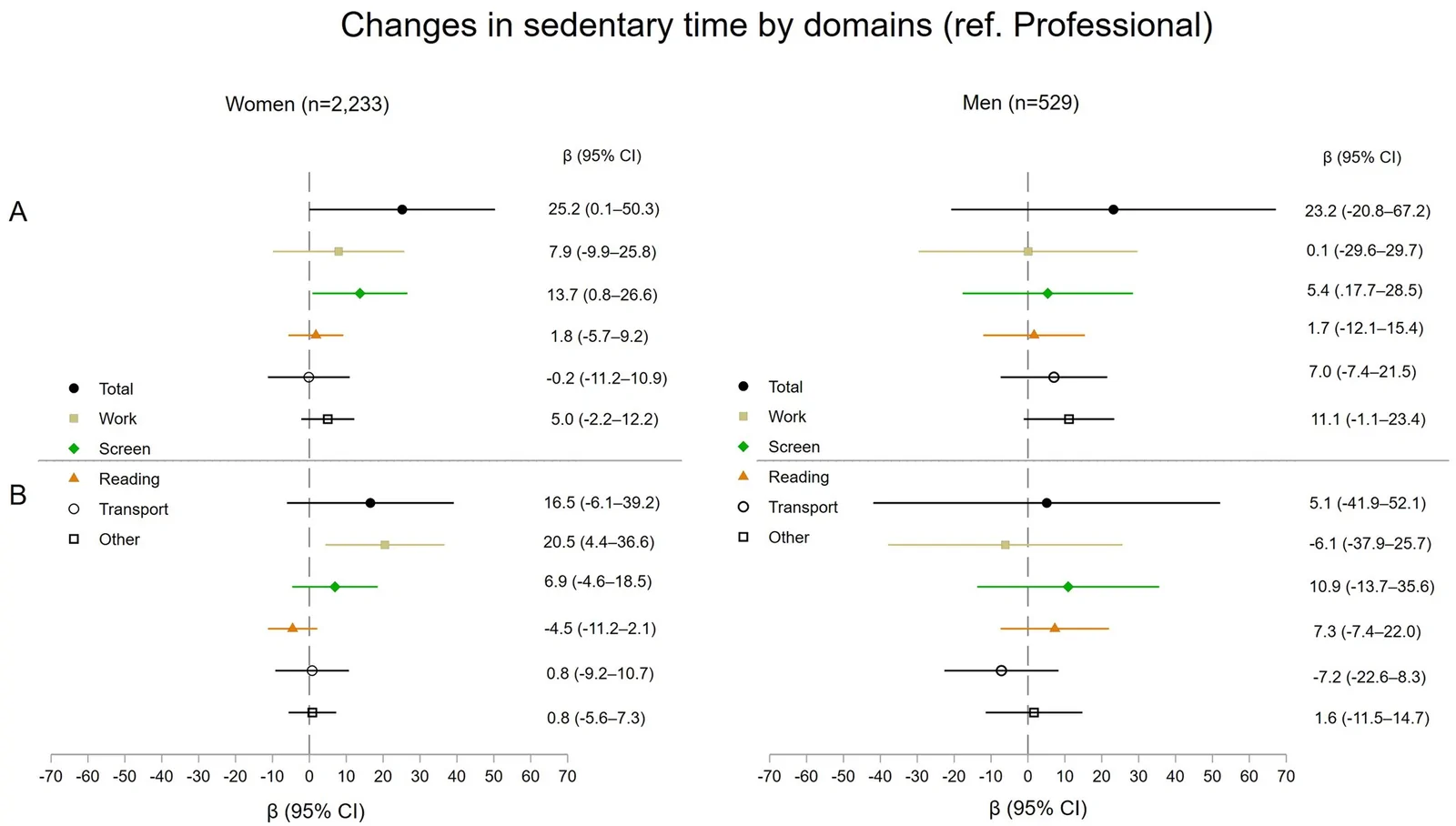
Changes in total and domain‐specific sedentary time (minutes/day) from 2017 to 2022 by occupational class and gender among the Helsinki Health Study participants (women *n = *2233; men *n = *529). Linear mixed‐effects models (random intercept for participant) estimated β‐coefficients (95% CIs) for the interaction between follow-up phase and occupational class (fixed effect), using the professional group (women *n = *625; men *n = *174) as reference; ‘A’ = manual/routine non-manual (women *n = *616; men *n = *201), ‘B’ = semi-professional (women *n = *992; men *n = *154). All models are adjusted for age.

Among women (Fig. 3), occupational class inequalities in total ST narrowed over the 5-year follow-up, whereas inequalities in leisure screen ST widened. Manual and routine non-manual employees had larger increases in total ST (β = 25 min, 95% CI: 0.1–50.3) and in leisure screen ST (β = 14 min, 95% CI: 0.8–26.6) when compared to professionals. Semi-professionals also showed a greater increase in work-related ST when compared to professionals (β = 21 min, 95% CI: 4.4–36.6), narrowing the occupational class differences. Despite this narrowing, professionals remained the group with the highest total ST in Phase 2. In contrast, the larger increase in leisure screen ST among manual and routine non-manual employees widened their differences compared to professionals by Phase 2 (Table S2).

After adjusting for sociodemographic and health‐related covariates, the associations remained largely unchanged—except that the increase in total ST among manual and routine non-manual employees no longer reached statistical significance (Table S3). When restricting the analysis to women whose occupational class remained constant over the 5-year follow-up (*n = *1732), the associations held and, in some domains, even showed slight strengthening when adjusted for age (Table S4). Among men, none of the associations could be statistically confirmed, although ST domains—particularly in the manual and routine non-manual group—paralleled those seen in women (Fig. 3, Table S3).

## Discussion

Our study examined the associations between occupational class and changes in total, work-related, leisure screen or reading, transportation, and other ST among young and early midlife Finnish employees over a 5-year follow-up (2017–22). During the follow-up, total daily ST increased among the study population, with notable increases in work-related and leisure screen ST. Women and men both showed significant increases, with generally larger changes among women. A key finding was the diverging pattern across ST domains: although occupational class difference in total ST narrowed somewhat over time, disparities in leisure screen ST widened, particularly between women in manual and routine non-manual classes occupations and women in professional class occupations. In addition, semi-professionals showed an increase in work-related ST compared to professionals, narrowing that specific domain gap by Phase 2. Among men, similar patterns were observed, but the associations did not reach statistical significance. Adjusting for covariates had little effect on the results, and findings remained consistent when restricting analyses to women whose occupational class did not change over time.

Overall ST increased notably over the 5-year follow-up, with an average increase of 46 minutes per day across the sample. This increase was shown by multiple domains: work-related ST, leisure screen ST, and reading, while ST in transportation decreased. These patterns may reflect a broader shift towards more sedentary behaviours in both occupational and non-occupational contexts. One possible explanation is changes in work practices, digitalization, and leisure habits, although these mechanisms were not directly assessed in our study.

Our findings are consistent with the FinHealth 2017 results for work-related and transportation domains but indicate lower levels of screen-based and other ST among Finnish adults of similar age [23]. A Finnish longitudinal study reported an average decrease of 26 minutes in total ST between 2007 and 2014 [9]. However, leisure screen ST increased 7–17 minutes, and lower occupational status was associated with greater increases in total ST.

A Canadian population-based study [11] found a slight overall increase in ST, but more importantly highlighted considerable individual variability, with many participants showing increasing, decreasing, or mixed patterns over time—consistent with previous cohort findings [24, 25]. A US study examining national trends reported a decline in ST between 2013 and 2020, which appeared to stall between 2021 and 2023, potentially reflecting the impact of the COVID-19 pandemic [10]. It is possible that pandemic-related restrictions also influenced the results of our study. The last 3 years of the follow-up period overlapped with the COVID-19 pandemic, which likely shaped both occupational and leisure-time ST. The expansion of remote work increased digital interactions, and restrictions on mobility may all have contributed to higher work-related and leisure screen ST, while reductions in commuting could partly explain lower transportation-related ST. Systematic reviews have reported that the pandemic was associated with a decrease in physical activity and an increase in sedentary behaviour [12, 13].

While the narrowing of occupational class differences in total ST might initially suggest a positive trend towards similarity, this alignment may instead point to a shared exposure to unfavourable behavioural risks. Recent studies have highlighted that even small increases in ST are associated with increased cardiometabolic risk, particularly when not offset by adequate physical activity [2, 26]. Therefore, the general rise in ST, regardless of occupational background, raises concern from a disease prevention perspective. Moreover, women exhibited overall higher increases in ST across most domains, although gender differences did not reach statistical significance. This aligns with previous evidence suggesting that women, particularly those in lower occupational positions, may face structural barriers to maintaining physically active routines during their leisure time [6, 27]. These may include unequal division of domestic responsibilities, reduced access to recreational opportunities, or psychosocial fatigue associated with emotionally demanding care work [28]. Consistent with this, our data also indicated lower levels of LTPA among women in lower occupational classes (data not shown). However, additional adjustment for LTPA did not change the associations between occupational class and ST, suggesting that occupational influences on ST extend beyond differences in LTPA.

The observed significant increase in daily work-related ST among women in the semi-professional class occupations may hypothetically relate to occupational composition and evolving work practices within this class these groups, although this interpretation cannot be confirmed in the present data. In the semi-professional class, the most common occupations included registered nurses and early childhood educators. Although traditionally considered physically active, these roles may have shifted towards more screen-based and administrative tasks over time, particularly with the increased digitalization of health and social care services [29]. The COVID-19 pandemic may have further amplified this shift by increasing remote meetings, online training, and documentation demands [13]. In contrast, professional class occupations—dominated by primary and secondary school teachers, psychologists, and general practitioners—may involve more varied movement throughout the workday, including standing, walking, or interaction-based tasks, which could limit increases in ST despite high occupational status. Furthermore, teachers and general practitioners mainly worked at present during Covid-19 pandemic in Finland. It is also possible that professionals have more autonomy over their schedules, enabling them to incorporate breaks or vary their work posture more freely than those in more task-constrained occupations [30].

At the same time, women in manual or routine non-manual class occupations (e.g. childcare workers and care workers) also showed significant increases in work-related ST, although their overall levels of occupational ST remained lower compared to the semi-professionals. This persistent gap may reflect structural differences in job tasks, with manual and care-related roles continuing to involve more physical movement and task-based activity despite some increase in documentation or screen use. The observed rise in occupational ST may have long-term health implications. Prior research has shown that individuals who predominantly sit at work have a higher mortality risk than those who do not [31], suggesting that even domain-specific sedentary behaviours may carry independent health consequences.

From the health inequality perspective, the widening disparity in leisure screen ST between women in manual or routine non-manual classes occupations and those in professional class roles may reflect broader social and structural inequalities in how leisure time is spent. Women in lower occupational classes may have fewer opportunities or resources for physically active or structured leisure [27, 32], leading to a greater reliance on passive screen-based activities during non-working hours. This increase in leisure screen ST may also be influenced by higher mental or physical fatigue from work [33], prompting recovery strategies that favour low-effort leisure. In contrast, professional class women—such as teachers, psychologists, and physicians—may be more likely to engage in diverse, non-sedentary leisure activities, which could help buffer against increases in screen-based ST [34]. These patterns suggest that occupational class may not only shape working life but also extend its influence into the structure and quality of leisure time. This trend is particularly concerning given previous study findings that have linked prolonged leisure screen time with increased all-cause mortality risk and physiological and mental health related disease risks [35–37]. As such, the disproportionate increase in leisure screen ST among women in lower occupational classes positions may indicate an emerging behavioural pathway contributing to future health inequalities.

### Methodological considerations

As the cohort comprised mainly women (81%) in the public sector, generalizability—to other sectors or the general population or men—is limited despite the participants reflecting the target population [18]. Although non-response analysis suggested that the respondents broadly represented the target population [17] and the response rate was acceptable, non-response for potential selection bias remains as a limitation. Additionally, the use of self-reported ST data may introduce the response bias leading to potential over- or underreporting or misclassification and recall bias [38]. In addition, the questionnaire used was not formally validated in this study population, although it closely resembles previously used domain-specific ST measures [39]. Another limitation is that unreported values in single ST domains were coded as zero when other domains were reported, which may have introduced some misclassification. The lack of weekend ST data could affect the primary results as ST patterns are known to differ between weekdays and weekends [40]. ST questionnaires, though not formally validated, are commonly used in epidemiological studies, whereas meta-analyses suggest that sensor-based methods provide more accurate estimates [19]. However, while sensor-based measurements offer objective data, they lack information on the setting of sedentary behaviour. Questionnaires also remain more feasible for large-scale epidemiological studies. In addition, an important consideration is whether the observed changes in ST truly reflect absolute changes in ST domains within the cohort or whether they are influenced by selection effects. Nonetheless, no significant baseline differences in ST were observed between the follow-up participants and dropouts, reducing concern for attrition bias (data not shown).

Our study has also several strengths. The continuous measure provided a concrete estimate of the magnitude of change in ST, capturing absolute increase in ST. Moreover, we examined a large employee cohort including both women and men and representing a wide range of occupations. We measured ST data at two time points and in different domains, investigating prospective relationships and changes in ST over the study period. We utilized a LMM approach to account for individual-level variability. This strengthens the model’s ability to estimate the effects of occupational class on ST over time with greater accuracy.

In conclusion, overall ST increased over a 5-year follow-up (2017–22), especially in work-related and leisure screen-based domains, with larger increases among women. Among women, lower occupational class was associated with greater increases in total and leisure screen ST, while semi-professionals showed increased work-related ST, narrowing the gap with professionals. Despite some convergence, professionals remained the most sedentary group. Future research should pair domain-specific ST with register or biomarker data and employ randomized and longitudinal designs to assess long-term impacts on health and healthcare use across work and leisure setting. Extending the follow-up beyond the 5-year period used in this study would allow a better understanding of whether increases in domain-specific sedentary behaviour—such as leisure screen or work-related ST—stabilize, continue to rise, or reverse over time and if the differences between occupational class will grow or diminish. This is particularly relevant in the context of societal transformations, including digitalization, remote work, and pandemic-related lifestyle changes. Such insights are essential for informing sustainable intervention strategies that target sedentary behaviour across socioeconomic groups. Our findings suggest that interventions should be tailored to occupational context and sedentary domains. Women in lower occupational classes, who showed the largest increases in total and leisure screen ST, may particularly benefit from public health strategies that support active leisure opportunities and reduce prolonged recreational sitting. In contrast, workplace-based interventions, such as sit-stand options, movement breaks, and organizational practices that reduce prolonged occupational sitting, may be particularly relevant professional and semi-professional occupations with high or increased work-related ST.

## Supplementary Material

ckag110_Supplementary_Data

## Data Availability

The Helsinki Health Study survey data (and the register data of the City of Helsinki) cannot be made publicly available due to strict data protection laws and regulations. The data can only be used for scientific research. More information on the survey data can be requested from the Helsinki Health Study research group (kttl-hhs@helsinki.fi).
