# Supplementary material for: Occupational class differences in daily sitting time among young and early midlife public sector employees—a follow-up study
Source: Eur J Public Health. 2026 Jun 24;36(4):ckag110. doi: 10.1093/eurpub/ckag110 (PMC13293066; doi:10.1093/eurpub/ckag110)
Supplement: ckag110_Supplementary_Data [file ckag110_supplementary_data.zip › ejph-2025-06-om-0536-File011.docx]

**Supplementary Table 4.** Change in daily sedentary time (minutes) between Phase 1 (2017) and Phase 2 (2022) across occupational classes for total, work-related, screen-based, reading, and other domains among women with stable occupational status during the Helsinki Health Study follow-up (n = 1,732). Estimates are β coefficients with 95% confidence intervals (CI); Model 1 is adjusted for age.

| **Women** (n=1,732) | Model 1 | |
| --- | --- | --- |
|  | Change in sedentary time | |
| Minutes / day | β | 95% CI |
| **Total sitting** |  |  |
| Overall sample (n=1,732) | 43.7 | 33.2–54.3 |
| Manual/routine non-manual (n=483) | 27.3 | 0.4–54.2 |
| Semi-professional (n=672) | 15.1 | -9.6–39.9 |
| Professional (n=577) | ref. |  |
| **Work** |  |  |
| Overall sample (n=1,303) | 34.0 | 26.8–41.2 |
| Manual/routine non-manual (n=363) | 1.2 | -17.0–19.4 |
| Semi-professional (n=484) | 20.8 | 3.9–37.7 |
| Professional (n=456) | ref. |  |
| **Leisure screen time** |  |  |
| Overall sample (n=1,732) | 19.8 | 14.4–25.1 |
| Manual/routine non-manual (n=483) | 14.4 | 0.7–28.1 |
| Semi-professional (n=672) | 2.2 | -10.4–14.8 |
| Professional (n=577) | ref. |  |
| **Leisure reading** |  |  |
| Overall sample (n=1,732) | 4.3 | 1.0–7.6 |
| Manual/routine non-manual (n=483) | 5.2 | -3.2–13.7 |
| Semi-professional (n=672) | -2.3 | -10.1–5.5 |
| Professional (n=577) | ref. |  |
| **Transport** |  |  |
| Overall sample (n=1,732) | -4.7 | -9.5–0.1 |
| Manual/routine non-manual (n=483) | 2.6 | -9.7–14.9 |
| Semi-professional (n=672) | 1.8 | -9.5–13.1 |
| Professional (n=577) | ref. |  |
| **Other** |  |  |
| Overall sample (n=1,732) | -1.2 | -4.2–1.7 |
| Manual/routine non-manual (n=483) | 5.3 | -2.3–12.8 |
| Semi-professional (n=672) | 0.3 | -6.7–7.2 |
| Professional (n=577) | ref. |  |
