# Supplementary material for: Occupational class differences in daily sitting time among young and early midlife public sector employees—a follow-up study
Source: Eur J Public Health. 2026 Jun 24;36(4):ckag110. doi: 10.1093/eurpub/ckag110 (PMC13293066; doi:10.1093/eurpub/ckag110)
Supplement: ckag110_Supplementary_Data [file ckag110_supplementary_data.zip › ejph-2025-06-om-0536-File008.docx]

**Supplementary Table 1**. A detailed description of the most common occupations in each occupational class by gender among the Helsinki Health Study participants (women n=2,233; men n=529).

| Occupational title | Frequency | Percent |
| --- | --- | --- |
| **Women** |  |  |
| **Manual/routine non-manual (n=616)** |  |  |
| Childcare worker | 210 | 34.2 |
| Practical nurse | 143 | 23.3 |
| Youth worker | 19 | 5.4 |
| Cook | <10 |  |
| **Men** |  |  |
| **Manual/routine non-manual (n=201)** |  |  |
| Firefighter | 29 | 14.5 |
| Practical nurse | 28 | 14.0 |
| Youth worker | 12 | 6.0 |
| Childcare worker | 10 | 5.0 |
| **Women** |  |  |
| **Semi-professional (n=992)** |  |  |
| Registered nurse | 395 | 39.8 |
| Early childhood teacher | 137 | 13.8 |
| Social care worker | 105 | 10.6 |
| Support worker | 83 | 8.4 |
| **Men** |  |  |
| **Semi-professional (n=154)** |  |  |
| Registered nurse | 44 | 28.6 |
| Support worker | 17 | 11.0 |
| Physiotherapist | 13 | 8.4 |
| Social care worker | <10 |  |
| **Women** |  |  |
| **Professional (n=625)** |  |  |
| School teacher | 266 | 42.6 |
| Social worker | 52 | 8.3 |
| Psychologist | 40 | 6.4 |
| General practitioner | 39 | 6.2 |
| **Men** |  |  |
| **Professional (n=174)** |  |  |
| School teacher | 59 | 33.9 |
| Project manager | <10 |  |
| General practitioner | <10 |  |
| Traffic engineer | <10 |  |
