# Supplementary material for: Occupational class differences in daily sitting time among young and early midlife public sector employees—a follow-up study
Source: Eur J Public Health. 2026 Jun 24;36(4):ckag110. doi: 10.1093/eurpub/ckag110 (PMC13293066; doi:10.1093/eurpub/ckag110)
Supplement: ckag110_Supplementary_Data [file ckag110_supplementary_data.zip › ejph-2025-06-om-0536-File010.docx]

**Supplementary Table 3.** Change in daily sedentary time (minutes) between Phase 1 (2017) and Phase 2 (2022) across occupational classes for total, work-related, screen-time, reading and other domains among women (n=2,233) and men (n=529) in the Helsinki Health Study. Estimates are β coefficients (95% confidence intervals, CI) from linear mixed model analyses. Model 1 is adjusted for age, Model 2 is adjusted for age, marital status, work status, body mass index, sufficient sleep, leisure-time physical activity, and self-rated health.

| **Women** (n=2,233) | Model 1 | | Model 2 | |
| --- | --- | --- | --- | --- |
|  | Change in sedentary time | | | |
| Minutes / day | β | 95% CI | β | 95% CI |
| **Total sitting** |  |  |  |  |
| Overall sample (n=2,233) | 47.4 | 38.1–56.8 | 48.1 | 38.7–57.6 |
| Manual/routine non-manual (n=616) | 25.2 | 0.1–50.3 | 24.7 | -0.6–50.0 |
| Semi-professional (n=992) | 16.5 | -6.1–39.2 | 17.9 | -4.8–40.6 |
| Professional (n=625) | ref. |  | ref. |  |
| **Work** |  |  |  |  |
| Overall sample (n=1,662) | 39.6 | 32.8–46.4 | 39.7 | 32.9–46.5 |
| Manual/routine non-manual (n=461) | 7.9 | -9.9–25.8 | 7.7 | -10.3–25.7 |
| Semi-professional (n=709) | 20.5 | 4.4–36.6 | 20.5 | 4.3–36.8 |
| Professional (n=492) | ref. |  |  |  |
| **Leisure screen time** |  |  |  |  |
| Overall sample (n=2,233) | 21.6 | 16.8–26.4 | 21.8 | 16.9–26.6 |
| Manual/routine non-manual (n=616) | 13.7 | 0.8–26.6 | 13.9 | 0.9–26.9 |
| Semi-professional (n=992) | 6.9 | -4.6–18.5 | 7.0 | -4.7–18.6 |
| Professional (n=625) | ref. |  |  |  |
| **Leisure reading** |  |  |  |  |
| Overall sample (n=2,233) | 2.5 | -0.2–5.3 | 2.5 | -0.2–5.3 |
| Manual/routine non-manual (n=616) | 1.8 | -5.7–9.2 | 1.6 | -5.9–9.1 |
| Semi-professional (n=992) | -4.5 | -11.2–2.1 | -4.4 | -11.2–2.3 |
| Professional (n=625) | ref. |  |  |  |
| **Transport** |  |  |  |  |
| Overall sample (n=2,233) | -4.9 | -9.1– -0.8 | -4.5 | -8.6– -0.4 |
| Manual/routine non-manual (n=616) | -0.2 | -11.2–10.9 | -0.4 | -11.5–10.7 |
| Semi-professional (n=992) | 0.8 | -9.2–10.7 | 1.7 | -8.2–11.6 |
| Professional (n=625) | ref. |  |  |  |
| **Other** |  |  |  |  |
| Overall sample (n=2,233) | -1.3 | -3.9–1.4 | -1.1 | -3.8–1.6 |
| Manual/routine non-manual (n=616) | 5.0 | -2.2–12.2 | 5.0 | -2.2–12.2 |
| Semi-professional (n=992) | 0.8 | -5.6–7.3 | 1.2 | -5.3–7.6 |
| Professional (n=625) | ref. |  |  |  |

| **Men** (n=529) | Model 1 | | Model 2 | |
| --- | --- | --- | --- | --- |
|  | Change in sedentary time | | | |
| Minutes / day | β | 95% CI | β | 95% CI |
| **Total sitting** |  |  |  |  |
| Overall sample (n=529) | 39.9 | 17.8–55.0 | 41.2 | 22.7–59.6 |
| Manual/routine non-manual (n=201) | 23.2 | -20.8–67.2 | 26.8 | -17.1–70.7 |
| Semi-professional (n=154) | 5.1 | -41.9–52.1 | 6.3 | -38.4–50.9 |
| Professional (n=174) | ref. |  | ref. |  |
| **Work** |  |  |  |  |
| Overall sample (n=479) | 31.1 | 18.6–43.6 | 32.1 | 19.7–44.6 |
| Manual/routine non-manual (n=178) | 0.1 | -29.6–29.7 | 2.8 | -26.6–32.2 |
| Semi-professional (n=136) | -6.1 | -37.9–25.7 | -6.1 | -37.6–25.3 |
| Professional (n=165) | ref. |  | ref. |  |
| **Leisure screen time** |  |  |  |  |
| Overall sample (n=529) | 15.7 | 6.0–25.4 | 15.8 | 6.1–25.5 |
| Manual/routine non-manual (n=201) | 5.4 | -17.7–28.5 | 5.5 | -17.6–28.7 |
| Semi-professional (n=154) | 10.9 | -13.7–35.6 | 10.9 | -13.8–35.6 |
| Professional (n=174) | ref. |  | ref. |  |
| **Leisure reading** |  |  |  |  |
| Overall sample (n=529) | 4.8 | -0.1–10.6 | 4.8 | -0.1–10.6 |
| Manual/routine non-manual (n=201) | 1.7 | -12.1–15.4 | 1.7 | -12.1–15.5 |
| Semi-professional (n=154) | 7.3 | -7.4–22.0 | 7.3 | -7.4–22.0 |
| Professional (n=174) | ref. |  | ref. |  |
| **Transport** |  |  |  |  |
| Overall sample (n=529) | -5.2 | -11.3–0.9 | -5.2 | -11.3–0.9 |
| Manual/routine non-manual (n=201) | 7.0 | -7.4–21.5 | 7.1 | -7.4–21.6 |
| Semi-professional (n=154) | -7.2 | -22.6–8.3 | -7.2 | -22.6–8.3 |
| Professional (n=174) | ref. |  | ref. |  |
| **Other** |  |  |  |  |
| Overall sample (n=529) | -3.7 | -8.8–1.5 | -3.3 | -8.5–1.8 |
| Manual/routine non-manual (n=201) | 11.1 | -1.1–23.4 | 12.1 | -0.1–24.3 |
| Semi-professional (n=154) | 1.6 | -11.5–14.7 | 1.6 | -11.4–14.6 |
| Professional (n=174) | ref. |  | ref. |  |
