# Supplementary material for: Occupational class differences in daily sitting time among young and early midlife public sector employees—a follow-up study
Source: Eur J Public Health. 2026 Jun 24;36(4):ckag110. doi: 10.1093/eurpub/ckag110 (PMC13293066; doi:10.1093/eurpub/ckag110)
Supplement: ckag110_Supplementary_Data [file ckag110_supplementary_data.zip › ejph-2025-06-om-0536-File009.docx]

**Supplementary Table 2.** Average daily sitting time (ST) minutes between occupational class across different ST domains among women (n=2,233) and men (n=529) of the Helsinki Health Study participants in 2017 (Phase1) and 2022 (Phase 2). The estimates are average marginal effect means with β values and 95% confidence intervals (CI) to provide mean ratios for sedentary time between phases. Model 1 is adjusted for age.

| **Women** (Model 1) | Phase 1 sedentary time | | Phase 2 sedentary time | | Change Phase 1–2 | |
| --- | --- | --- | --- | --- | --- | --- |
| Minutes / day | β | 95% CI | β | 95% CI | β | 95% CI |
| **Total sitting (n=2,233)** |  |  |  |  |  |  |
| Overall sample | 378 | 369–387 | 426 | 417–434 | 47.4 | 38.1–56.8 |
| Manual/routine non-manual | 344 | 327–360 | 402 | 385–419 | 58.3 | 31.7–85.0 |
| Semi-professional | 381 | 368–394 | 431 | 418–444 | 49.7 | 28.7–70.7 |
| Professional | 408 | 391–424 | 441 | 424–458 | 33.1 | 6.7–59.6 |
| **Work (n=1,662)** |  |  |  |  |  |  |
| Overall sample | 200 | 193–207 | 239 | 232–246 | 39.6 | 32.8–46.4 |
| Manual/routine non-manual | 142 | 129–155 | 178 | 165–191 | 36.6 | 17.5–55.7 |
| Semi-professional | 211 | 201–221 | 260 | 250–271 | 49.1 | 33.7–64.6 |
| Professional | 237 | 225–250 | 266 | 253–278 | 28.7 | 10.1–47.2 |
| **Leisure screen time (n=2,233)** |  |  |  |  |  |  |
| Overall sample | 115 | 111–119 | 137 | 133–141 | 21.6 | 16.8–26.4 |
| Manual/routine non-manual | 123 | 116–131 | 152 | 144–159 | 28.5 | 14.8–42.1 |
| Semi-professional | 112 | 107–118 | 134 | 128–140 | 21.7 | 10.9–32.4 |
| Professional | 111 | 103–118 | 126 | 118–133 | 14.8 | 1.2–28.3 |
| **Leisure reading (n=2,233)** |  |  |  |  |  |  |
| Overall sample | 37 | 35–39 | 39 | 37–42 | 2.5 | -0.2–5.3 |
| Manual/routine non-manual | 34 | 30–38 | 40 | 36–44 | 5.8 | -2.0–13.7 |
| Semi-professional | 37 | 34–40 | 37 | 33–40 | -0.5 | -6.7–5.7 |
| Professional | 40 | 36–44 | 44 | 40–48 | 4.1 | -3.8–11.9 |
| **Transport (n=2,233)** |  |  |  |  |  |  |
| Overall sample | 57 | 54–60 | 52 | 49–55 | -4.9 | -9.1– -0.8 |
| Manual/routine non-manual | 59 | 53–65 | 54 | 48–60 | -5.4 | -17.1–6.4 |
| Semi-professional | 58 | 54–63 | 54 | 49–58 | -4.5 | -13.7–4.8 |
| Professional | 52 | 46–58 | 47 | 41–52 | -5.2 | -16.9.–6.4 |
| **Other (n=2,233)** |  |  |  |  |  |  |
| Overall sample | 21 | 19–23 | 20 | 18–22 | -1.3 | -3.9–1.4 |
| Manual/routine non-manual | 19 | 15–23 | 21 | 17–25 | 2.0 | -5.6–9.6 |
| Semi-professional | 22 | 18–25 | 19 | 16–23 | -2.2 | -8.2–3.8 |
| Professional | 22 | 18–26 | 19 | 15–23 | -3 | -10.6–4.6 |

| **Men** (Model 1) | Phase 1 Sitting | | Phase 2 Sitting | | Change Phase 1–2 | |
| --- | --- | --- | --- | --- | --- | --- |
| Minutes / day | β | 95% CI | β | 95% CI | β | 95% CI |
| **Total sitting (n=529)** |  |  |  |  |  |  |
| Overall sample | 454 | 436–472 | 494 | 476–511 | 39.9 | 21.4–58.3 |
| Manual/routine non-manual | 427 | 398–456 | 480 | 451–509 | 52.8 | 8.0–97.5 |
| Semi-professional | 476 | 442–509 | 510 | 477–543 | 34.7 | -16.5–85.8 |
| Professional | 465 | 434–497 | 495 | 464–526 | 29.6 | -18.5–77.7 |
| **Work (n=479)** |  |  |  |  |  |  |
| Overall sample | 214 | 201–228 | 245 | 232–259 | 31.1 | 18.6–43.6 |
| Manual/routine non-manual | 178 | 156–199 | 211 | 189–232 | 32.9 | 2.2–63.6 |
| Semi-professional | 223 | 199–248 | 250 | 225–274 | 26.7 | -8.4–61.8 |
| Professional | 246 | 224–269 | 279 | 257–301 | 32.8 | 0.9–64.7 |
| **Leisure screen time (n=529)** |  |  |  |  |  |  |
| Overall sample | 145 | 136–153 | 160 | 152–169 | 15.7 | 6.0–25.4 |
| Manual/routine non-manual | 154 | 141–168 | 170 | 157–184 | 15.9 | -7.6–39.4 |
| Semi-professional | 146 | 131–161 | 167 | 152–183 | 21.4 | -5.4–48.3 |
| Professional | 132 | 118–147 | 143 | 128–157 | 10.5 | -14.7–35.6 |
| **Leisure reading (n=529)** |  |  |  |  |  |  |
| Overall sample | 35 | 30–39 | 40 | 35–44 | 4.8 | -0.1–10.6 |
| Manual/routine non-manual | 33 | 26–40 | 37 | 30–44 | 3.7 | -10.3–17.7 |
| Semi-professional | 38 | 30–46 | 47 | 39–55 | 9.4 | -6.6–25.3 |
| Professional | 34 | 27–42 | 36 | 29–44 | 2.1 | -13.0–17.1 |
| **Transport (n=529)** |  |  |  |  |  |  |
| Overall sample | 56 | 51–61 | 51 | 46–56 | -4.9 | -9.1– -0.8 |
| Manual/routine non-manual | 60 | 52–68 | 61 | 53–69 | 1.3 | -13.4–16.0 |
| Semi-professional | 65 | 56–74 | 52 | 43–61 | -13.0 | -29.8–3.5 |
| Professional | 44 | 35–52 | 38 | 29–47 | -5.8 | -21.6–10.0 |
| **Other (n=529)** |  |  |  |  |  |  |
| Overall sample | 24 | 20–28 | 20 | 17–24 | -3.7 | -8.8–1.5 |
| Manual/routine non-manual | 22 | 16–28 | 25 | 19–31 | 2.8 | -9.7–15.2 |
| Semi-professional | 29 | 22–36 | 22 | 15–30 | -6.7 | -21.0–7.5 |
| Professional | 22 | 15–29 | 14 | 7–20 | -8.4 | -21.8–5.0 |
