# Supplementary material for: Occupational class differences in daily sitting time among young and early midlife public sector employees—a follow-up study
Source: Eur J Public Health. 2026 Jun 24;36(4):ckag110. doi: 10.1093/eurpub/ckag110 (PMC13293066; doi:10.1093/eurpub/ckag110)
Supplement: ckag110_Supplementary_Data [file ckag110_supplementary_data.zip › ejph-2025-06-om-0536-File007.docx]

Supplementary File 2. Additional methods Supplement: Survey questions used in the study

*Note: Most participants responded in Finnish or Swedish. The following is an English translation of the survey questions, which was also provided as an optional language version in the questionnaire.*

BACKGROUND INFORMATION

1. Are you a
   - man
   - woman
2. What is your year of birth? ________
3. What is the highest level of examination or qualification that you have attained?
   - elementary school, primary school, lower secondary school, middle school or less
   - vocational qualification
   - upper secondary school / matriculation / A-levels
   - Bachelor’s degree (for example UAS or university)
   - Master’s degree (for example UAS or university)
   - doctoral degree
4. Are you currently mainly:
   - in full-time work
   - in part-time work
   - on family leave (parental leave, maternity leave, paternity leave etc.)
   - a student
   - long-term (over 6 months) sick leave
   - a recipient of rehabilitation allowance
   - on disability pension
   - unemployed
   - other
5. What is your marital status?
   - single (never married)
   - cohabiting
   - married or in a registered partnership
   - separated or divorced
   - widowed

EXERCISE

1. Next, we will be asking about physical activity during your leisure and commuting time over the past 12 months. We have divided physical activities in four levels of exertion. First, estimate the exertion level of the physical activities you are engaged in. Then, estimate how often you engage in a physical activity equivalent to each level of exertion during one week rounded to closest 15 minutes (e.g. 02 hours and 45 minutes).
   1. During your leisure time

| **Strenuousness of**  **exercise** | **Hours** | **Minutes** |
| --- | --- | --- |
| Equivalent to walking | ________ | ________ |
| Equivalent to brisk walking | ________ | ________ |
| Equivalent to light running (jogging) | ________ | ________ |
| Equivalent to brisk running | ________ | ________ |

- 1. During your commute

| **Strenuousness of**  **exercise** | **Hours** | **Minutes** |
| --- | --- | --- |
| Equivalent to walking | ________ | ________ |
| Equivalent to brisk walking | ________ | ________ |
| Equivalent to light running (jogging) | ________ | ________ |
| Equivalent to brisk running | ________ | ________ |

1. How much time on average do you sit each weekday rounded to closest 15 minutes? Enter 0, if you spend no time sitting at all.

|  | **Hours** | **Minutes** |
| --- | --- | --- |
| At home watching television or sitting in front of the computer | ________ | ________ |
| At home reading | ________ | ________ |
| Sitting in a vehicle (e.g. car, train) | ________ | ________ |
| At work | ________ | ________ |
| Elsewhere | ________ | ________ |

HEIGHT

1. How tall are you? ________cm
2. How much do you weigh? ________kg (round to the nearest kilogram)

SLEEP

1. Do you feel that you get enough sleep?
   - yes, almost always
   - yes, often
   - rarely or hardly ever
2. In general, would you say your health is:
   - excellent
   - very good
   - good
   - fair
   - poor
